# Contractility Measurements of Human Uterine Smooth Muscle to Aid Drug Development

**DOI:** 10.3791/56639

**Published:** 2018-01-26

**Authors:** Sarah Arrowsmith, Peter Keov, Markus Muttenthaler, Christian W. Gruber

**Affiliations:** ^1^Harris-Wellbeing Preterm Birth Research Centre, Department of Cellular and Molecular Physiology, Institute of Translational Medicine, University of Liverpool; ^2^School of Biomedical Sciences, The University of Queensland; ^3^Faculty of Chemistry, Institute of Biological Chemistry, University of Vienna; ^4^Institute for Molecular Bioscience, University of Queensland; ^5^Center for Physiology and Pharmacology, Medical University of Vienna

**Keywords:** Medicine, Issue 131, Physiology, pharmacology, myometrium, human uterus, contraction, organ bath, peptide, oxytocin, vasopressin, drug discovery

## Abstract

Discovery and characterization of novel pharmaceutical compounds or biochemical probes rely on robust and physiologically relevant assay systems. We describe methods to measure *ex vivo* myometrium contractility. This assay can be used to investigate factors and molecules involved in the modulation of myometrial contraction and to determine their excitatory or inhibitory actions, and hence their therapeutic potential *in vivo*. Biopsies are obtained from women undergoing cesarean section delivery with informed consent. Fine strips of myometrium are dissected, clipped and attached to a force transducer within 1 mL organ baths superfused with physiological saline solution at 37 °C. Strips develop spontaneous contractions within 2–3 h under set tension and remain stable for many hours (>6 h). Strips can also be stimulated to contract such as by the endogenous hormones, oxytocin and vasopressin, which cause concentration-dependent modulation of contraction frequency, force and duration, to more closely resemble contractions in labor. Hence, the effect of known and novel drug leads can be tested on spontaneous and agonist-induced contractions.

This protocol specifically details how this assay can be used to determine the potency of known and novel agents by measuring their effects on various parameters of human myometrial contraction. We use the oxytocin- and V_1a_ receptor antagonists, atosiban and SR49059 as examples of known compounds which inhibit oxytocin- and vasopressin-induced contractions, and demonstrate how this method can be used to complement and validate pharmacological data obtained from cell-based assays to aid drug development. The effects of novel agonists in comparison to oxytocin and vasopressin can also be characterized. Whilst we use the example of the oxytocin/ vasopressin system, this method can also be used to study other receptors and ion channels that play a role in uterine contraction and relaxation to advance the understanding of human uterine physiology and pathophysiology.

**Figure:**
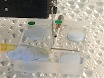


## Introduction

The goal of drug discovery is to produce novel, potent, and highly selective ligands which generate a therapeutic response by activating or inhibiting cellular signaling pathways. This requires an appropriate assay system in which to test lead compounds with reliable, robust, and relevant outcomes^1^. Pharmacological techniques such as ligand-receptor binding and functional assays often employ heterologous cell-based systems, engineered to overexpress receptors which otherwise would not be present^2^. Whilst these techniques provide valuable insights for receptor pharmacology and early drug development, the data obtained may not reflect a true *in vivo *scenario. It is therefore important that pharmacological data from cell-based assays are also validated in physiologically relevant models.

Uterine smooth muscle (myometrium) constitutes the muscular layer of the uterus which is responsible for contractions during labor that efface and dilate the cervix and deliver the fetus^3^. Contraction of myometrium is spontaneous; it does not require hormonal or nervous input to contract^4^. Contractions are brought about by spontaneous depolarization of the myometrial cell membrane which leads to the opening of voltage-operated Ca^2+^-channels (L-Type channels) and influx of Ca^2+^ into the cell^5^. Calcium complexes with calmodulin and activates myosin light chain kinase which in turn phosphorylates myosin enabling cross bridge cycle formation with actin and contraction. Relaxation is typically mediated by de-phosphorylation of myosin by myosin phosphatase and a reduction in the concentration of Ca^2+^ via its extrusion from the cell and/or sequestering into the sarcoplasmic reticulum (SR)^5^,^6^,^7^.

Several methods have been developed to study myometrial function and dysfunction, from *in vivo* internal and external tocography and intrauterine pressure catheters, to the generation of transformed or immortalized cells of myometrial origin^8^,^9^,^10^,^11^,^12^. Whilst cell culture systems can detect whether a substance can act at the cellular level, strips of tissues within organ baths can be used as tools to measure functional responses of whole tissues to pharmacological reagents. The traditional organ bath is typically a large heated glass chamber which is capable of holding between 5 and 50 mL of physiological saline (PSS). Strips within these large chambers normally require aeration with oxygen and large volumes of PSS. Strips of tissue are dissected and suspended within the bathing chamber and attached to a force transducer which measures changes in tension, such as during a contraction. Strips of myometrium from both humans and animals including, guinea pig^13^, mouse^14^, rat^15^, rabbit^16^, and others^17^,^18^, have been used by a number of research groups to examine many questions relating to myometrial physiology and pathology, including preterm and dysfunctional labors. For example, myometrial strips have been used to identify factors which regulate and modify myogenic activity^19^,^20^,^21^, determine organelle function such as the SR^22^, as well as investigating modulators of ion channels^23^,^24^,^25^,^26^, pumps and exchangers^27^ to determine their role in myometrial physiology.

This *ex vivo* technique allows for the assessment of tissue contraction performance and the direct effect of different agents on the parameters of contraction to be measured including, contraction force (strength), frequency, and duration, as well as the integration of these values, to generate an index of the total work done (mean integral force or area under the curve, AUC). As the isolated tissue strip preparation constitutes a model of more than one cell type, the physiological response of the whole tissue can be measured. The organ bath and isolated tissue strip are therefore a useful tool in providing the bridge between cell culture work and whole animal/*in vivo* work. Hence, in the field of drug discovery, the effects of novel agents in terms of their excitatory (*i.e.*, stimulating) or inhibitory (*i.e.*, relaxation) potential *in vivo *can be more closely assessed. This technique was successfully used in the development of the oxytocin- and V_1a_-receptor (OTR and V_1a_R) antagonist atosiban as a tocolytic agent to inhibit preterm labor contractions and delay preterm birth. *In vitro* testing of atosiban on myometrial strips found the antagonist able to significantly reduce oxytocin (OT)-induced contractions^28^,^29^,^30^. Importantly these studies helped to validate the translational value of atosiban and generated the proof-of-concept data needed to take it forward to clinical trials^31^,^32^,^33^,^34^. Atosiban is now widely used as the drug of choice to delay labor in Europe. Similar proof-of-concept studies were carried out for the oxytocin analog carbetocin to show its therapeutic potential in preventing post-partum haemorrhage^35^,^36^,^37^. Other lead compounds currently under development with this assay include retosiban (GSK221149A)^38^ and nolasiban (data unpublished). These methods have also been used successfully to compare between patient groups^39^,^40^,^41^,^42^,^43^,^44^,^45^,^46^,^47^ and examine for intra-species differences.

Here we describe the use of isolated *ex vivo* tissue strips from pregnant human myometrium within small (1 mL) custom-made organ baths to illustrate how this method can be used to complement and validate pharmacological data obtained from cell-based assays. Biopsies were largely obtained from women undergoing pre-labor elective Cesarean Section (CS) delivery as they are planned surgery and hence are easier to schedule biopsy collection, have easy access to the myometrium, and tissues will not have been exposed to any uterotonic stimulants or relaxants prior to surgery. However, biopsies can also be obtained from women undergoing unplanned (emergency) CS delivery in labor providing there is sufficient time to fully consent the patient. Most biopsies are obtained during surgery from the lower uterine segment at the site of surgical incision, however it is also possible to obtain samples from the upper segment^48^,^49^. In a few cases following vaginal delivery, punch biopsies from the placental bed have also been obtained^50^. However, this is not the most conventional route and the amount of myometrial tissue retrieved is small. Non-pregnant myometrium can be obtained from pre- or post-menopausal women undergoing hysterectomy for benign gynecological conditions. A full thickness biopsy is sampled post-pathology examination, from the lower corpus away from the cervix, and myometrium is taken from the middle of the uterine wall avoiding the serosal and endometrial surfaces.

## Protocol

Appropriate institutional, ethical review board and safety approval for experiments with human tissues must be in place before working with any human tissues. All work described herein received approval from the Local Research Ethics committee (Liverpool East, REC Ref 10/H1002/49) and institutional review boards of the Research and Development Department, Liverpool Women's Hospital and University of Liverpool.

NOTE: All biopsies described in this protocol were obtained from women undergoing pre-labor elective CS delivery at Liverpool Women's Hospital and each woman gave written informed consent to participate.

### 1. Solutions

1. Prepare modified Krebs physiological saline solution (PSS) with the following composition: 154 mM NaCl, 5.6 mM KCl, 1.2 mM MgSO_4_, 7.8 mM glucose, 10.9 mM HEPES, and 2.0 mM CaCl_2_. NOTE: The volume to be made is determined according to the number of tissue baths in operation, the flow rate of the system (1 mL/min), and estimated length of experiment including equilibration and washout time.
2. Adjust pH to 7.4 using 4 M NaOH.

### 2. Tissue Bath Set Up

1. Preheat the tissue bath system reservoir to ~45 °C using a recirculating water bath set at 55-60 °C. NOTE: The water reservoir in the base of the apparatus is heated to ~45 °C, which after allowing for heat exchange with the PSS in the peristaltic tubing running through, ensures the PSS in the tissue bath is at 37 °C. Some adjustment to set temperatures may be needed to ensure the temperature of the PSS in the tissue bath reaches 37 °C. This will vary depending on the apparatus used and set flow rates.
2. Manually switch on any other necessary equipment including the amplifier, data acquisition and recording system, and suction pumps.
3. Position each peristaltic feeder tube (one tube per bath) around the rollers of the peristaltic pump head. Secure with the retaining stops and by tightening the compression cams and locking keys around the tubes. Place the free ends of the peristaltic feeder tubes into a 1 L container of PSS and start the pump to allow the PSS to continually perfuse into the tissue baths.
4. Ensure the suction pumps are operating correctly and that the level of solution in the bath is constant such that the rate of flow into the bath equals the rate of removal. The level of PSS in the tissue bath can be adjusted by manipulating the depth of the suction tube in the bath using the modeler's clay.
5. Calibrate force transducers by placing a known weight (equivalent to 1 millinewton (mN, unit of force)) onto the transducer hook and recording the deflection detected by the acquisition software. NOTE: Values can be adjusted in the software such that the values recorded are automatically converted to mN negating the need to perform any further conversions during analysis. Calibration of force transducers must be performed before tissue mounting.

### 3. Tissue Preparation and Dissection of Strips

1. Collect biopsies of pregnant human myometrium (1–2 cm^3^) at CS after delivery of the baby and placenta. Obtain (typical) full thickness biopsies in which both the perimetrium (outer serosa layer of the uterus) and decidua (innermost layer of uterus) are present. Use only myometrial tissue (central muscle layer). See Figure 1A**-B**.
  1. Remove (essential) decidua and any adherent fetal membranes (if present) as these tissues are known to produce substances which can alter myometrial contractility. NOTE: In surgery, samples are placed into Hanks Balanced Salt Solution (HBSS) or fresh PSS and stored at 4 °C. Ideally tissues should be used within 12-16 h of surgery, however studies have shown no detriment to contractility after 18 h of collection with storage at 4 °C^51^. Appropriate controls must be performed to confirm tissue viability and function after prolonged storage at 4 °C. The samples here are taken from the upper edge of the lower uterine segment incision site and not from the upper segment. The lower and upper segments of the uterus have been shown to contract similarly^48^, hence lower segment biopsies are considered to be a good reflection of upper segment myometrial activity.
2. Prepare the dissection area and required instruments including: large dissection scissors, small Vannas dissecting scissors, two dissecting forceps, dissection pins, and aluminum tissue clips, around a dissection microscope with both fixed and zoom magnification.
3. Place the biopsy sample on a clear silastic-based dissection dish (see **Table of Materials**) filled with PSS and carefully orientate the biopsy so that the serosa and decidua edges are identified Figure 1B.
4. Use pins to secure the biopsy to the base of the dish. Ensure the tissue remains hydrated with PSS throughout the dissection process.
5. Manually switch on the microscope light source and inspect the tissue under the microscope at 10x magnification to identify regions of myometrium free from scar tissue, serosa, decidua, and any adherent fetal membranes if present.
6. Perform blunt dissection using large dissection scissors to separate two adjacent tissue layers, revealing two planes or sheets of muscle. NOTE: It is often easier to find a small pocket between tissue layers to begin the separation but care must be taken to avoid small vessels at the biopsy edge as they can be mistaken for 'pockets'.
7. Place dissecting pins on each corner of the tissue to secure it. Inspect the sheets of muscle looking for regions of muscle with fibers running in parallel. NOTE: Region identification can be aided by following the direction of small capillaries perfusing through the tissue. Gentle pulling at the tissue edge can also help identify the direction in which the fibers are traveling.
8. Cut away strips of tissue ~ 2 mm wide x 8 mm long x 1 mm thick along the longitudinal axis aligned with the direction of the muscle fibers using small dissection scissors. NOTE: Care must be taken to only hold the tissue by the initial cut edge to avoid damage.
9. Repeat the process to dissect out the number of required strips for the experiment.
10. Pin each strip at both ends to straighten and secure to the dish, taking care not to over stretch the tissue.
11. Attach aluminum tissue clips at each end so that the tissue between them is ~ 5 mm long and carefully cut away any excess tissue (Figure 1C).
12. Transfer to a clean dish filled with PSS ready for mounting.

### 4. Mounting the Tissues in the Baths

1. Transfer the strips to the experimental tissue baths. Mount the strips horizontally rather than vertically (as in traditional organ baths) (Figure 2B).
2. Attach one end of each strip by the clip to the force transducer, which measures the tissue contraction, and the other to a fixed hook both within the tissue chamber. NOTE: The capacity of the tissue bath is ~1 mL which is large enough to ensure the strips are completely submerged in the bath.
3. Ensure the strips are at the base of each hook in the bath.
4. Open the recording software by double clicking on the software icon.
5. Adjust the recording for each channel so that it is in view in the channel window.
6. Adjust the Y axis scale to read between 0-10 V (equivalent to 0-10 mN post calibration) by right clicking on the Y axis, selecting 'scale', and inputting minimum and maximum values of 0 and 10, respectively. Select 'OK'. Repeat for each channel recording.
7. Press 'record' on the software to begin live recording. NOTE: If strips are not secured to the base of each hook, they could slip during contraction which will appear as a 'notch' on the recording. The set tension will change and therefore contractions post-slippage may differ.
8. Stretch the tissue in each bath by manually turning the micromanipulators attached to each transducer. Follow the upward tissue movement from baseline on the screen and continue to turn the micromanipulators until the baseline tension reaches 0.2 g (~2 mN). NOTE: The tissue will immediately begin to relax (noted by a drop in baseline tension), typically reaching a steady tension of between 0.5–1 mN. This defined tension has been optimized for tissue strips of this size in our experimental conditions. If other sized tissues are to be used, it is essential that appropriate length-tension relationship investigations are performed to optimize the resting force to be applied.
9. Allow the tissues to equilibrate for ~ 2 h until spontaneous contractions arise. NOTE: a small elevation in baseline tension is usually observed and is a good indication of tissue viability and that it will be contractile.

### 5. Challenging the Tissue with 40 mM Potassium (High K^+^)

1. If no spontaneous contractions occur following 2 h of mounting, challenge the strips with a high potassium salt solution in which potassium is elevated to 40 mM by isosmotic substitution of NaCl for KCl. For the high K^+^, add the following (in mM): 119.6 NaCl, 40 KCl,1.2 MgSO_4_, 7.8 glucose, 10.9 HEPES, and 2.0 CaCl_2_. NOTE: In smooth muscle such as myometrium, application of high K^+^ causes contraction by indirectly opening voltage operated calcium channels leading to maximal influx of Ca^2+^ into the tissue cells. High K^+^ can therefore be used as a measure to test the general index of tissue integrity as well as achieving a measure of maximal tissue response.
  1. Place the feeder tube for the bath containing the strip to be challenged into a glass laboratory bottle containing high K^+^ for 1-2 min.
  2. Return the feeder tube to the container of PSS.
  3. Where a response to high K^+^ is achieved, continue monitoring the strip for a further 1 h. If there is no contractile response to high K^+^ or no spontaneous activity elicited post high K^+^ response, discard the strip. NOTE: The experimenter also needs to be aware of the dead space (time taken for solution to reach the tissue bath) in the feeder tubing before assessing the response to high K^+^. This may take 2-3 min and will depend on flow rate.
  4. To ensure complete washout of the high K^+^ from the bath and feeder tubes, which could affect subsequent maneuvers, wait until spontaneous contractions return to pre high K^+^ amplitude before proceeding further with the experiment.

### 6. Testing the Effects of Known and Novel Compounds on Myometrial Contraction

NOTE: Experiments to test the effect of novel drugs and reagents on myometrial function can be performed on spontaneous (Figure 3A) or agonist-stimulated contractions (Figure 3B**-C**). For agonist stimulated contractions, OT or arginine vasopressin (VP) is added to the PSS to give a concentration of 0.5 nM and is used throughout the experiment (Figure 3B**-C**). The concentration of OT has been optimized for this assay as it provides contractions which are phasic in nature^40^,^52^. Concentrations of agonists greater than this can lead to long lasting, sustained (tonic) contractions (see Ref^41^,^44^ for examples) under which the effect of various agents is difficult to assess and the experimental time frame needs to be greatly extended to accommodate the increased duration of individual contractions and reduction in frequency. In this example experiment, OT or VP is added to stimulate contractions so that antagonism by known OTR and V_1a_R antagonists, atosiban and SR49059, or by our novel compound [D-Arg8]-inotocin ([D-Arg8]INT), can be assessed.

1. Prepare concentrations of the test compound(s) in PSS (with or without 0.5 nM OT/VP) diluted at a minimum of 1/1,000 dilution, ensuring a wide range of concentrations are included such as those that do not elicit a response to concentrations that exceed the maximal response.
2. Prepare an equivalent set of dilutions involving equal volumes of vehicle (*e.g.*, dimethyl sulfoxide, acetonitrile, or distilled water). NOTE: It is often easiest to prepare the highest concentration, *i.e.*, 10^-6^ M (from a stock of 10^-3^ M) and then perform serial dilutions along a logarithmic scale (*e.g.*, 10^-6^^-^10^-10^ M). The volume to be prepared depends upon the time of application, flow rate of the apparatus, and number of strips to be examined, *e.g.*, for a 25 min application of reagent and a flow rate of 1 mL/min, a minimum of 25 mL of each concentration per tissue bath is required.
3. Apply the first concentration of compound to the tissue by placing the feeder tubing for the tissue bath into a glass laboratory bottle containing the reagent at the desired concentration. NOTE: This should be done once a steady baseline of spontaneous or OT/VP-stimulated contractions is achieved.
4. Apply the corresponding vehicle control solution in the same way to a second bath.
5. Record the time of application (*i.e.*, when the feeder tubing was changed).
6. Repeat the process for each concentration by sequentially placing the feeder tubing into the glass laboratory bottle containing the next concentration in the series and repeat until all concentrations have been applied. The application of each concentration can be between 15 and 30 min but should be consistent for each concentration applied and between experiments. Those involving OT or VP-stimulated contractions tend to require the longer application periods (*e.g.*, 25 min) to account for the reduction in contraction frequency observed under stimulation. Preliminary experiments to determine the optimal application time should be performed beforehand with any new reagent being tested.
7. Return the feeder tube to PSS (with or without OT/VP) to washout.
8. Click 'stop' to end the live recording. Immediately save the data to an appropriate folder and export a version as a '.mat' file.

### 7. Data Analysis

NOTE: Data capture and analysis can be performed by any number of commercially available data acquisition software packages. See **Table of Materials** for details of software used in this protocol. For an accurate assessment of contractile activity, the parameters of contractions to be measured include: i) amplitude of contraction, ii) frequency of contraction, iii) duration of contraction, and iv) mean force integral (Figure 4). Mean force integral is the equivalent to area under the contraction curve and is therefore an index of the total work done by the tissue strip in a given time. Some or all of the parameters can be analyzed. As a minimum, it is recommended that the mean integral force and amplitude of contraction are analyzed^53^. In the experiments described here we measured changes in amplitude of contraction and area under the curve.

1. Import the.mat file into the analysis software.
2. Adjust the column corresponding to the X axis to reflect the experimental time, taking into account the sampling interval frequency. Experiments are typically recorded at 10 Hz corresponding to 10 samples/s or 600 samples/min.
3. Plot the data as an X-Y co-ordinate graph using "Plot | Line" function.
4. Zero the baseline of contraction using the 'translate vertical' function on the software.
5. Select an appropriate control period. NOTE: This is the period of time immediately preceding the application of the first concentration of regent but equal to the duration of application of reagent (Figure 4A). For instance, if the application of drug X is for 25 min, use the 25 min preceding the first application of drug X as the control.
6. Read off the Y axis, record the amplitude (force) of contraction at the max peak of each contraction occurring in the timeframe selected, and calculate a mean value.
7. Measure the duration of contraction at 50% of this maximum peak by reading off the X axis at the start and end of each contraction and record a mean value.
8. Count the number of contractions occurring in the time frame to generate a value for frequency.
9. Use the 'integration' function to calculate AUC (in arbitrary units, a.u.) for the period of time selected. NOTE: To record AUC accurately, it is essential that the baseline of the contractions is set at zero on the Y axis.
10. Sequentially move through each of the concentrations and record the different parameters of contraction.
11. Set the control data as 100% and express the values obtained under each concentration of reagent as a percentage of this control *i.e.*, values for stimulation should be >100% whilst relaxation should be <100%. NOTE: Normalizing the data in this way should allow the user to compare results across strips and pharmacological treatments.
12. Repeat for each experiment and transfer the data to a graphical package.

## Representative Results

Using this model, the response to various agonists and antagonists of contraction as well as novel agents of known or unknown function can be examined and quantified. Standard pharmacological parameters such as EC_50_ and IC_50_ values can be calculated when reagents are used in a wide range of concentrations, *e.g.*, 10^-5^–10^-9^ M and added in increasing concentrations along a logarithmic scale.

**Oxytocin Receptor Antagonist Concentration-response Experiments** In this experiment, paired strips of human myometrium were cut as described above and shown in Figure 1, mounted in the tissue baths as depicted in Figure 2, and allowed to equilibrate to produce stable contractions of equal amplitude and frequency. Strips were then exposed to the endogenous OTR agonist, OT (0.5 nM) to stimulate contractions. After a period of stable activity under OT (typically 45 min), atosiban was applied to one strip in increasing concentrations along a logarithmic scale (10^-9^–10^-6^ M). The second strip was left in OT alone as time-control. An example of the response to atosiban can be seen in Figure 5A. Taking the contractions immediately preceding the first concentration of atosiban as control (100%), the amplitude and AUC for each concentration applied was calculated as shown in Figure 4. For time-control experiments, the time-equivalent of experimental maneuvers was measured. The data were then plotted and curves fitted using the non-linear regression function in a graphical software package (Figure 5B-**C**). In terms of calculating the inhibitory effect, the relative potency of atosiban was calculated by measuring the IC_50_ which is the concentration causing half maximal (50%) inhibitory response. The same can be done for agonists or stimulators of contraction. In this case the potency of the compound is calculated from the EC_50_ (the concentration causing half maximal stimulatory response).

**Investigating the Response of Novel Compounds and Testing Their Receptor Selectivity** We used this physiologically relevant model of *ex vivo* human myometrial contractions to examine the antagonistic effects of a newly synthesized compound, [D-Arg8]-inotocin ([D-Arg8]INT) on contractions stimulated with either the native V_1a_R agonist, VP, or the native OTR agonist, OT. We used this assay to validate the receptor selectivity of [D-Arg8]INT, which had been previously determined by pharmacological cell-based methods to be an antagonist at the V_1a_R but not at the OTR^54^.

In this experiment, human myometrial strips were exposed to 0.5 nM VP or 0.5 nM OT for around 1 h to stimulate contractions as shown in Figure 3, prior to adding our novel compound [D-Arg8]INT in increasing concentrations (Figure 6A and Figure 6C). This was then compared to the commercially available, known V_1a_R antagonist, SR49059 (Figure 6B). Figure 6A illustrates concentration dependent decreases in VP-stimulated human myometrial contractions with increasing concentrations of [D-Arg8]INT. The data for amplitude of contraction and AUC for each concentration are summarized in Figure 6Aii**-iii**. The effect is similar to that shown for increasing concentrations of the known V_1a_R inhibitor, SR49059, shown in Figure 6Bi**-iii**. The selectivity of [D-Arg8]INT towards the V_1a_R but not towards the OTR is demonstrated by the fact that [D-Arg8]INT does not decrease human myometrial contractions which have been stimulated with OT (Figure 6C) as amplitude and AUC remained stable (Figure 6Cii**-iii**).

**Figure:**
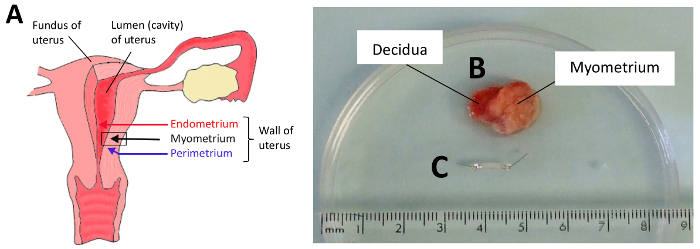

**Figure 1: Human myometrial biopsy dissection. **(**A**) A diagram of the human uterus showing the three tissue layers that comprise the uterine wall. The innermost layer is the endometrium (decidua in pregnancy, red arrow), the middle layer is the myometrium (muscular layer, black arrow) which generates contractions, and the outermost layer is the perimetrium (or serosal membrane, blue arrow) which forms a protective coat around the uterus. The region of interest for biopsy sampling is depicted by the black rectangle. An example biopsy from a pregnant woman taken during caesarean section is shown in (**B**) with the decidua and myometrial layers highlighted (serosa not visible). It is essential that the different tissue layers are identified so that strips of myometrium are correctly dissected for experimentation. An example strip of myometrium which has been dissected and clipped is shown in (**C**). Typically, 2–6 strips are cut and clipped as shown. Please click here to view a larger version of this figure.

**Figure:**
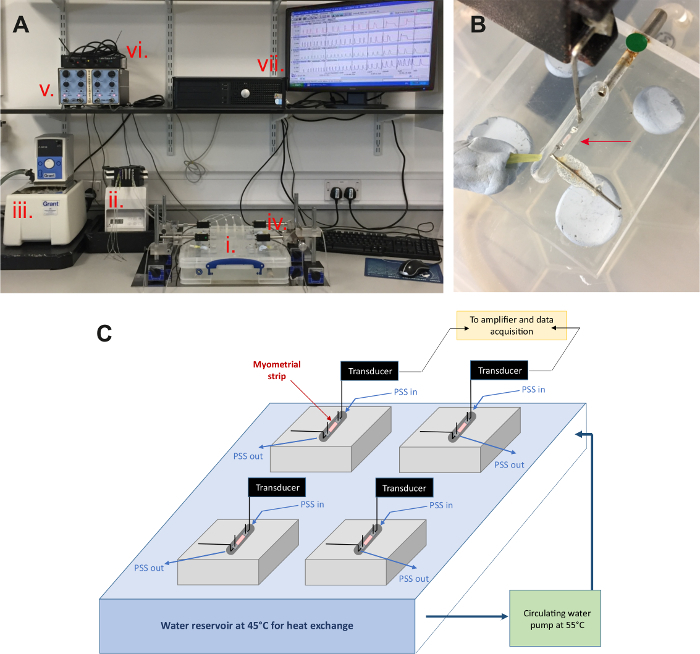

**Figure 2: Multi-organ bath experimental set up used to measure contractions of human myometrium.** (**A**) Strips of myometrium are placed into small (1 mL) organ bath chambers placed on top of a heated reservoir (i) and are superfused with physiological saline solution (PSS) by way of a peristaltic pump (ii). The reservoir is maintained at a set temperature by way of a circulating water bath (iii). Each strip is attached to a force transducer (iv), which records changes in tension during contraction. This is amplified by a transbridge amplifier (v) and converted into a digital signal (vi), which is recorded on a computer system (vii) running the associated acquisition software. (**B**) Enlarged image of an organ bath chamber with a strip of human myometrium *in situ* (red arrow), bathed in PSS with one end attached to a force transducer and the other to a fixed hook.**(C**) Schematic overview of the set up. Tissue chambers filled with PSS are continually perfused with warmed PSS at 37 °C which is via heat exchange with a heated water reservoir beneath the baths (kept at 45 °C) and a circulating water pump set at 55 °C. Please click here to view a larger version of this figure.

**Figure:**
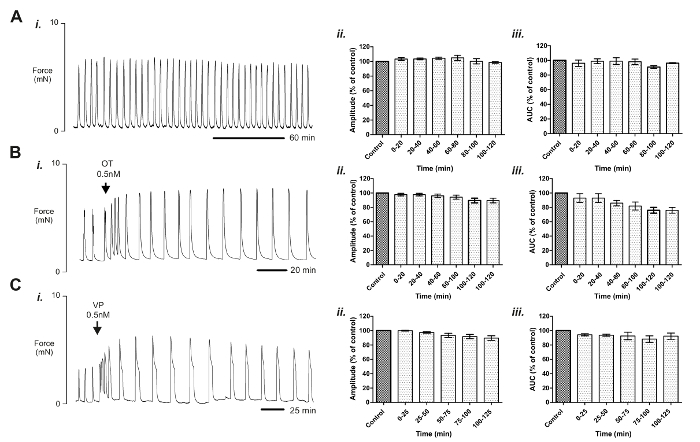

**Figure 3: Spontaneous and agonist-stimulated contractions of human myometrium *in vitro.*** In agonist-free conditions, spontaneous contractions remain stable for over 3 h of recording without significant loss of amplitude or area-under-the-curve (AUC) (**Aii**, **Aiii**), demonstrating the robustness of this model for investigating the application of various agents on spontaneous contraction. After establishing stable, spontaneous contractions, 0.5 nM oxytocin (**B**, OT) or vasopressin (**C**, VP) was added to the physiological saline solution (PSS). Contractions under stimulation also remain stable for a number of hours without significant loss of contraction amplitude (**Bii**, **Cii**) or AUC (**Biii**, **Ciii**) enabling the effect of various contractile agents to be investigated in the presence of myometrial agonists. Data are presented as mean ± standard error of the mean (SEM). Note, for agonist-stimulated contractions (**B**, **C**), the control period (100%) is taken after the first 45 min of application of the agonist, once contractions have stabilized. In all cases, strips were superfused with PSS at 37 °C, pH 7.4 (Reproduced from Arrowsmith *et al.*^40^ with permission from *Reproductive Sciences* and Di Giglio *et al.*^54^ with permission from *Scientific Reports* under the Creative Common Open Access license). Please click here to view a larger version of this figure.

**Figure:**
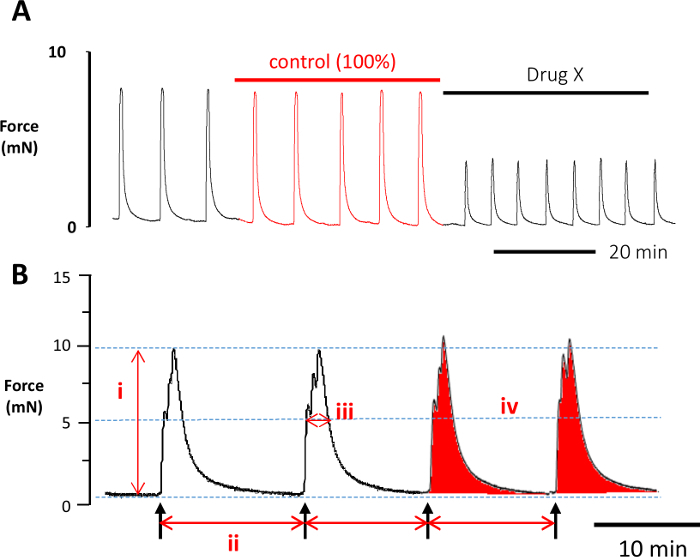

**Figure 4: Data analysis.** (**A**)****An appropriate control period shown in red was determined by selecting contractions immediately preceding the application of the test compound (Drug X). This control period is also equal in time to the application of Drug X (*e.g.*, 40 min in this example). Once measured the values for the control period are set as 100%. All subsequent measurements are then expressed as a percentage of control. (**B**) There are 4 different parameters of contraction that can be measured: (i) amplitude of contraction which measures contraction strength (force, mN), (ii) frequency of contraction which measures rate of contraction, (iii) duration of contraction which is measured at half maximal peak of contraction, and (iv) area under the curve (also known as force integral, arbitrary units) which gives a measure of overall work done. Please click here to view a larger version of this figure.

**Figure:**
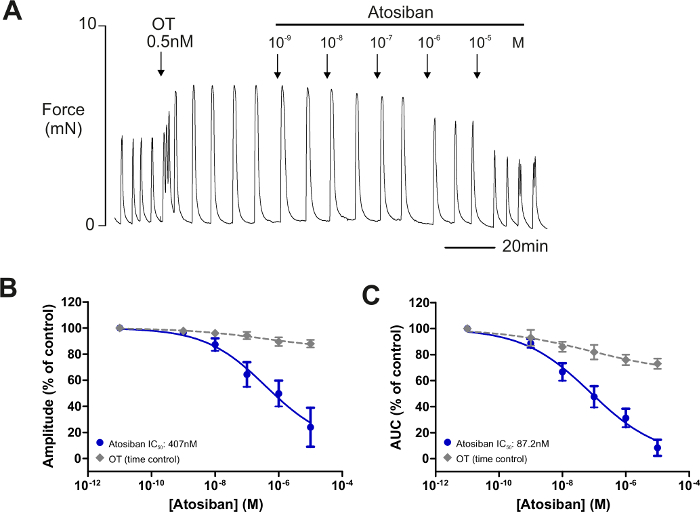

**Figure 5: Recording the antagonistic effect of atosiban on oxytocin-induced contractions in human myometrium.** Once spontaneous contractions were established, contractions were stimulated with the oxytocin receptor agonist, oxytocin (OT). Contractions were allowed to stabilize under stimulation for a further 45 min. (**A**) The V_1a_ and OT receptor antagonist, atosiban was then applied in increasing concentrations along a logarithmic scale (10^-9^-10^-5^M). The contractions during the period preceding the first concentration of atosiban were measured and taken as control (100%). The activity under each subsequent concentration was measured and expressed as a percentage of control. The same was performed for strips exposed to OT alone using the time-equivalent of experimental maneuvers. Data were plotted in graphical software: (**B**, **C**) show concentration-response curves for the antagonistic effect of atosiban (blue) and appropriate dilution of vehicle (gray, time control) on OT-induced myometrial contraction amplitude and area under the curve (AUC), respectively. Data are presented as the mean ± standard error of the mean (SEM) percentage of amplitude and AUC of control activity (before the application of atosiban). IC_50_ values were then calculated which give the concentration at which the half maximal inhibitory (50%) response for amplitude of contraction and force integral (AUC) is achieved (Reproduced from Arrowsmith *et al.*^40^ with permission from *Reproductive Sciences*). Please click here to view a larger version of this figure.

**Figure:**
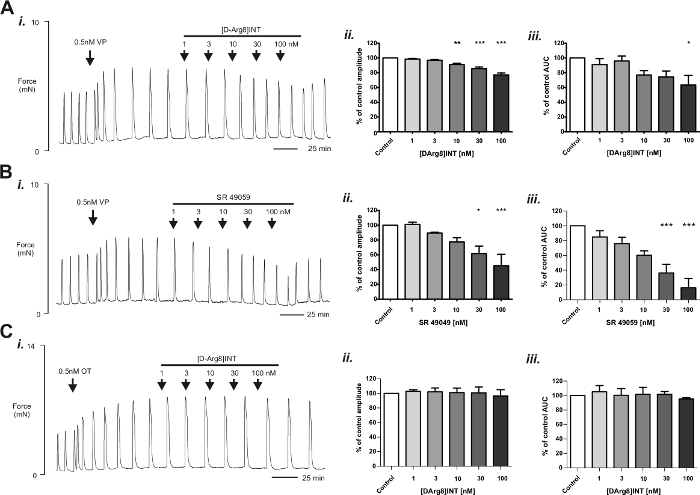

**Figure 6: Testing the effect and receptor selectivity of a novel compound on human myometrial contraction.** After spontaneous contractions of human myometrium were established, contractions were stimulated with either the vasopressin receptor agonist, vasopressin (VP) (**Ai, Bi**) or the oxytocin receptor agonist, oxytocin (OT) (**Ci**). The effect of [D-Arg8]-inotocin ([D-Arg8]INT) and the commercially available V_1a_R antagonist, SR49059 on contractions stimulated by VP was assessed by applying increasing concentrations of the compounds. Typical responses are shown in (**Ai**, **Bi**). The associated analyzed data for amplitude and area under the curve (AUC) are shown in (**ii**) and (**iii**), respectively where the effect has been expressed as percentage of control activity (100%). Both [D-Arg8]INT and the known V_1a_R antagonist, SR49059 caused a dose dependent decrease in amplitude and AUC, supporting the role of [D-Arg8]INT as a V_1a_R receptor antagonist in human myometrium. In contrast, [D-Arg8]INT did not affect contractions stimulated by OT (**Ci-iii**), hence similarly to our findings from cell based assays, [D-Arg8]INT also shows selectivity towards the V_1a_R in human myometrium (Data reproduced from Di Giglio *et al.*^54^ with permission from *Scientific Reports* under the Creative Common Open Access license). Please click here to view a larger version of this figure.

## Discussion

Whilst most drug development is intended for the treatment of human disorders, most basic research is primarily performed in animal tissues. Here, we describe methods to investigate *ex vivo* contractions of human myometrium obtained from surgery which can be used to address a number of important questions related to uterine physiology and pathology, as well as to validate functional responses to known and novel pharmacological agents to aid drug development. In particular, we highlight the use of this assay to investigate the antagonistic response of a known OTR and V_1a_R competitive antagonist, atosiban on OT-induced pregnant myometrial contractions, as well as to determine the response and receptor selectivity of a newly developed selective V_1a_R antagonist, [D-Arg8]INT. We demonstrate that important pharmacokinetic parameters such as EC_50_ and IC_50_ can be calculated, which align well with the cell-based pharmacology data.

Using multiple strips simultaneously allows for direct comparison of multiple agent effects, competition experiments with antagonists, and appropriate time and vehicle controls. As strips are often prepared in groups of 2, 4, or 8, this technique provides moderate throughput, enabling the testing of 2–4 compounds at 6–8 (cumulative) concentrations (per biopsy). This method also provides real-time data so that the effects can be assessed quickly and protocols can be adjusted. In addition, this technique can be used to test any compound of interest and has been used successfully by a number of research groups focused on myometrial physiology and in drug discovery. Besides analyzing pure compounds as described in this paper, the uterine contractility model has also been and can be successfully utilized to screen for novel uterotonic compounds from mixtures, such as herbal preparations from traditional medicine^7^,^55^,^56^.

Although this model is technically robust and shows good reproducibility, it does have some limitations: Dissection can be difficult for those unfamiliar with the tissue or using dissection equipment, hence new users will require some time to optimize tissue preparation and protocols. It should also be noted that human myometrium differs considerably in appearance to other models such as rat and mouse. Most rodent uteri are composed of two tube-like uterine horns, each complete with an ovary at one end and joined at the cervix. Each horn has clearly defined longitudinal and circular muscle layers, which can be easily separated at dissection whilst the different fiber types in human myometrium are often intertwined forming a 'mesh'. In addition, the contraction profiles of human myometrium are very different to rodents. Most notably, contractions in human myometrium are less frequent but longer in duration. Experimental time frames for working with human myometrium are therefore often much longer than rodent models. Differences in receptor expression between species can also contribute to quite marked differences in responses to agonists and this should be borne in mind if extrapolating results across species.

There are also number of steps that need consideration to maximize output from this system. Critical steps include preservation of tissue viability such as taking care when handling tissues to avoid any unnecessary damage during dissection or when mounting. A skilled eye is required to dissect fine strips of uterine muscle, ensuring the orientation of muscle fibers is in the longitudinal direction and following the plane of the tissue, as well as avoiding scar tissues, decidua, and small vessels. To aid orientation purposes once the specimen is in the laboratory, it is possible to add a tag (such as small surgical stitch) at the time of collection to one side of the biopsy to delineate the serosal edge from the decidual edge.

Tissues should be kept at a steady 36–37 °C during experimentation as tissue function is subject to temperature fluctuations. This can be achieved with a robust air conditioning unit within the laboratory. Constant perfusion of warmed PSS ensures the temperature is maintained as well as the flushing out of waste products from contraction. Temperature within the organ baths can be altered by changing flow rate or adjusting the water bath temperature directly. The small bath size compared to traditional 5-50 mL baths ensures a relatively rapid turnover of PSS and washout of reagents. The miniaturized organ bath as described here, also reduces the volume of PSS and reagents of interest needed, thus minimizing costs and sparing precious, newly developed chemicals. In addition, owing to the small bath size and using a HEPES-based buffer, this system does not require oxygenation *e.g.*, by bubbling the PSS with carbogen. Standardization of tension applied to the strips is also important. For strips of this size (5 mm x 2 mm x 1 mm), this should be approximately 2 mN (equivalent to ~0.2 g). Alternative methods include application of a high potassium solution to induce maximal contraction and stretching to half of this maximum contraction. It must be noted however, that tension applied *in vivo* may differ.

The main challenges include obtaining tissues from human subjects, but although taxing, human tissues clearly represent the most physiologically relevant (and rewarding) model for studying uterine contraction in human disease and drug discovery. The isolated tissue strips however, do not necessarily equate to tissue *in vivo* as they are exempt for example, of hormonal and nervous input which, although not essential for contraction, will modulate contractions *in vivo*. This assay however provides the opportunity to analyze myometrial contractions in a controlled manner, separated from such influences. It also allows for the effect of factors such as hormonal control of contraction (*e.g.*, via OT, VP, prostaglandins, *etc.*) to be investigated, providing clues to the regulation of myometrial function. As tissues are obtained from different women, there is naturally some variation in the spontaneous contractile profiles between specimens. Hence it is often necessary to perform experiments on a large number (~n = 10) of samples to minimize the variation in some datasets^53^. This is most important when comparing contractile activity between different patient groups. Normalizing the response of agonists and antagonists to control activity (*i.e.*, expressing as a percentage of control activity or high K^+^) reduces some of this variation. In addition, to reduce inter-strip variation, data can be normalized to strip cross sectional area by measuring their length and weight post experimentation^41^. This is particularly useful when comparing contraction patterns between different patient groups.

Limitations of this technique also include access to fresh tissues which requires good working relationships with hospital staff, especially theater staff and those involved in the consent process. Ethical permissions from the local Research Ethics Committee and Institute or hospital review board also need to be in place. Collection of human myometrium is most likely performed during CS delivery, when the donor is undergoing surgery. The biopsy is taken from the same uterine incision site made for delivering the baby and therefore the patient does not need to undergo any further additional procedures. Theater staff and the surgeon performing the biopsy need to be made aware of the subsequent use of the tissues and that they are not to be placed into fixative solutions such as for sending to a pathology department. The long experimental timeframe is another consideration. Experiments with human tissues take many hours (typically >6 h) (as opposed to 2–3 h for a similar experiment in rat or mouse uterus), owing to the slower frequency of contraction and the 2–3 h lag time between tissue mounting and establishment of spontaneous contractions. However, as we have shown, human tissue contractions are robust, and when established can contract for many hours without significant fatigue^40^.

This system also allows other challenges of *in vivo *work to be overcome including the ability to test pharmaceutical reagents on pregnant tissue. This technique can be easily extrapolated to other species including mouse whereby initial results can be confirmed before proceeding further in whole animal studies. Controlled changes in temperature, composition of the superfusate (PSS), and pH can be made easily to mimic different scenarios *in vivo* and analyze the effect of these changes on compound behavior. The basic principles of measuring isometric tension in myometrial strips can also be expanded to measuring simultaneous changes in intracellular calcium concentration or pH by the use of fluorescent Ca or pH (H^+^) indicators and equipment to detect and record fluorescence^57^,^58^,^59^,^60^.

Overall, the human myometrium represents a robust and physiologically relevant model to characterize and validate novel therapeutic compounds in drug discovery — both pure compounds and mixtures. We have provided examples of its use in drug discovery with respect to the OT/VP system and focused on OTR and V1_a_R antagonists to show how this model can be used to determine compound efficacy and potency at defined targets and validate ligand selectivity. However, it should be borne in mind that this technique can be used to study any target of interest or pathway leading to myometrial contraction (or relaxation), as well as to aid drug discovery of new targets and pathways, and advance our understanding of myometrial physiology and pathophysiology.

## Disclosures

The authors have nothing to disclose.
